# Age and sun exposure-related widespread genomic blocks of hypomethylation in nonmalignant skin

**DOI:** 10.1186/s13059-015-0644-y

**Published:** 2015-04-16

**Authors:** Amy R Vandiver, Rafael A Irizarry, Kasper D Hansen, Luis A Garza, Arni Runarsson, Xin Li, Anna L Chien, Timothy S Wang, Sherry G Leung, Sewon Kang, Andrew P Feinberg

**Affiliations:** Center for Epigenetics, Johns Hopkins University School of Medicine, Rangos 570, 855N. Wolfe St, Baltimore, MD 21205 USA; Dana-Farber Cancer Institute, CLSB 11007, 450 Brookline Ave, Boston, MA 02215 USA; Department of Biostatistics and Institute for Genetic Medicine, Johns Hopkins University School of Medicine, 615N. Wolfe St, E3527, Baltimore, MD 21205 USA; Department of Dermatology, Johns Hopkins University School of Medicine, CRB II Room 204, 1550 Orleans Street, Baltimore, MD 21287 USA; Department of Medicine, Johns Hopkins University School of Medicine, Baltimore, MD 21205 USA

## Abstract

**Background:**

Aging and sun exposure are the leading causes of skin cancer. It has been shown that epigenetic changes, such as DNA methylation, are well established mechanisms for cancer, and also have emerging roles in aging and common disease. Here, we directly ask whether DNA methylation is altered following skin aging and/or chronic sun exposure in humans.

**Results:**

We compare epidermis and dermis of both sun-protected and sun-exposed skin derived from younger subjects (under 35 years old) and older subjects (over 60 years old), using the Infinium HumanMethylation450 array and whole genome bisulfite sequencing. We observe large blocks of the genome that are hypomethylated in older, sun-exposed epidermal samples, with the degree of hypomethylation associated with clinical measures of photo-aging. We replicate these findings using whole genome bisulfite sequencing, comparing epidermis from an additional set of younger and older subjects. These blocks largely overlap known hypomethylated blocks in colon cancer and we observe that these same regions are similarly hypomethylated in squamous cell carcinoma samples.

**Conclusions:**

These data implicate large scale epigenomic change in mediating the effects of environmental damage with photo-aging.

**Electronic supplementary material:**

The online version of this article (doi:10.1186/s13059-015-0644-y) contains supplementary material, which is available to authorized users.

## Background

Aging is the greatest single risk factor for cancer, cognitive decline, frailty, and immunological dysfunction [1], yet consistent genomic alterations related to aging have been elusive. For example, few genetic variants regulating human life span have been identified [2,3]. At the same time, there is a growing realization that environmental factors are major contributors to aging and age-associated illness. Epigenetics is the study of chemical modifications of the genome, heritable by cell progeny, and it has been an attractive target for studies of aging and environmentally influenced disease. Several groups have shown differences in DNA methylation - a covalent modification of cytosine at CpG dinucleotides - in peripheral blood samples and other tissues with increasing age [4-6]. Some of these differences are possibly confounded by changes in cell type distribution with aging [7], but many are likely real as they are seen across multiple cell types. Studies of identical twins have shown markedly divergent patterns of DNA methylation in whole blood over the lifespan, suggesting an environmental component to epigenetic change with age [8], and the epigenetic drift hypothesis [9].

The skin as a model of aging offers the advantage of studying the influence of environmental factors by virtue of its direct exposure to the sun. The superficial layer of epidermis (approximately 60 μm) interfaces more directly with the outside world than the deeper dermal layer. Even penetration of solar ultraviolet (UV) radiation is mostly in the epidermis [10]. Furthermore, skin affords the ability to compare the effects of intrinsic and extrinsic (environmental) aging through the comparison of chronically sun-exposed (for example, forearm and face) and sun-protected skin (for example, upper inner arm) in the same individual. Both layers offer a relatively homogenous system for analyzing DNA methylation; the epidermis especially is composed of around 95% keratinocytes [11]. Histological changes associated with aging and sun exposure in human skin have been extensively studied. The primary histopathological changes associated with aging and sun exposure are independent of cell type change: in some studies of the epidermal layer, increased thickness is associated with sun exposure, decreased thickness is associated with aging. Within the dermal layer, loss of collagen and altered fibroblast morphology is associated with both chronological aging and sun exposure [12,13]. Despite the lack of large cell type shifts, both extrinsic and intrinsic skin aging are associated with broad changes in gene expression, with many similar pathways differentially regulated in each. Pathways associated with intrinsic aging in sun-protected skin are seen to be amplified by chronic environmental exposure in sun-exposed skin [14,15]. We therefore hypothesized that epigenetic changes might mediate the changes associated with environmental exposure in aging skin.

A previous study of DNA methylation in aging skin tissue was limited technologically. This study used the Infinium HumanMethylation27 BeadChip, which measures 27,000 CpG sites, focused on dense CpG regions termed CpG islands [16]. It showed little change related to skin aging (hypermethylation at 0.38% of sites) and even less change associated with sun exposure (hypomethylation at 0.05% of sites) [16]. We and others have recently observed widespread differentially methylated regions (DMRs) across the genome that distinguish tissues (t-DMRs), stages of stem cell reprogramming (r-DMRs), and cancer (c-DMRs). Most of these alterations are either at regions near but not in CpG-dense islands, termed CpG island shores, or distal from both islands and shores (the 'open seas') [17-20]. These distal regions were discovered to be large blocks, corresponding to heterochromatin regions termed large organized chromatin lysine-modifications (LOCKs) or nuclear lamin-associated domains (LADs), and these large blocks show substantial hypomethylation in cancer [20,21]. Almost none of these regions is represented on the Infinium HumanMethylation27 BeadChip, and thus were not included in the previous study. A more recent study used whole genome bisulfite sequencing (WGBS) to examine methylation in aging skin more comprehensively, but they looked only at samples from one body site, the inner forearm, of each individual and thus were not able to characterize the interaction between intrinsic and extrinsic aging in these samples [22].

In order to more fully examine methylation in human skin samples affected by age and sun-exposure, we performed array-based DNA methylation analysis using the Infinium HumanMethylation450 BeadChip array, which includes islands, shores, most known c-DMRs, t-DMRs, and r-DMRs and probes within the 'open sea' regions identified as part of the hypomethylated blocks in cancer [23]. We applied recently developed algorithms [24] capable of identifying differences in large blocks as have previously been detected in cancer samples using WGBS, and we confirmed these results directly with WGBS of additional samples. Here we describe profound changes in DNA methylation associated with combined aging and sun exposure, involving 670 Mb of the genome, and including large blocks similar to cancer. In human subjects, these changes are progressive with quantitative measures of skin aging. These same blocks are observed to be hypomethylated in squamous cell carcinoma samples.

## Results

We undertook a comprehensive genome-wide analysis of DNA methylation in human skin to test specifically for alterations associated with age or with sun exposure, and the potential relationship between these regions. Ten younger individuals (<35 years old) and 10 older individuals (>60 years old) each submitted one sun-protected biopsy specimen from the upper inner arm, as well as one sun-exposed specimen from either the dorsal forearm or crow’s feet (lateral epicanthus) (donor information in Additional file 1). Donors were carefully selected to exclude individuals with active skin conditions or individuals using topical medications (exclusion criteria detailed in methods). As methylation and sun-exposure effects both vary with race, we limited this sample set to Caucasian donors. In addition to tissue biopsies, donors volunteered health history information. For each donor, a dermatologist evaluated their degree of apparent skin aging in both sun-exposed and sun-protected regions using two established scales.

In order to study more homogenous tissue samples, each punch biopsy was mechanically separated into epidermis and dermis following overnight incubation with dispase, a procedure associated with unappreciable fibroblast contamination in cultured epidermal sections [25], and analyzed separately. In previous work, epidermal sections separated in this manner show the same age- and sun exposure-related methylation changes as epidermal sections separated by suction blister, indicating the dispase separation does not have significant effects on the methylome [16]. To confirm uniformity of our technique among sample groups, we sectioned a subset of dispase separated epidermal layers and performed hematoxylin and eosin staining. Examination of stained sections showed separation below the basal layer for all age and sun exposure groups, consistent with previous histological analysis of dispase separated epidermis (Additional file 2) [26]. To characterize methylation genome-wide, we used the Infinium HumanMethylation450 bead chip (450k), which includes most CpG islands and shores, additional regions shown to be differentially methylated in cancer and development, and other functionally important regions [23].

**Figure 1:**
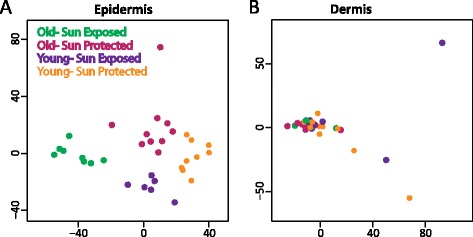
DNA methylation in epidermal but not dermal samples clusters by age and sun exposure. **(A)** In epidermis, DNA methylation segregates old versus young individuals, and also segregates sun-exposed and sun-protected anatomical regions, shown by multidimensional scaling of pairwise distances derived from methylation levels assayed on the HumanMethylation450 BeadChip (450k). **(B)** In dermis, DNA methylation does not segregate samples by age or anatomical region, shown by multidimensional scaling of pairwise distances derived from methylation levels assayed on the 450k.

We classified the samples from each skin layer into four groups: younger sun-protected (Y-pro), younger sun-exposed (Y-exp), older sun-protected (O-pro), and older sun-exposed (O-exp). To obtain an overview of the genome-scale differences among these eight groups, we performed principal component analysis and created scatter plots of the first two principal components. As expected given the established differential methylation between tissue types [17], the greatest differences were between dermis and epidermis, supporting the experimental rationale of dissociating those two tissues. There was also clear separation between samples obtained from either sun-protected or sun-exposed arm and from the face, indicating that this anatomical difference had to be considered during subsequent analysis. When a principal component analysis was repeated with just the epidermal arm samples, there was striking separation among the four groups, with the greatest difference between O-exp and Y-pro (Figure 1A). The sun-exposed samples obtained from the face cluster separately, but maintain the separation between old and young. Principal component analysis on dermal arm samples did not show separation related to age and exposure (Figure 1B), suggesting that the number of differences would be much less than in epidermis, consistent with the previous studies [16].

### Hypomethylated blocks associated with aging and sun exposure in epidermis

**Figure 2:**
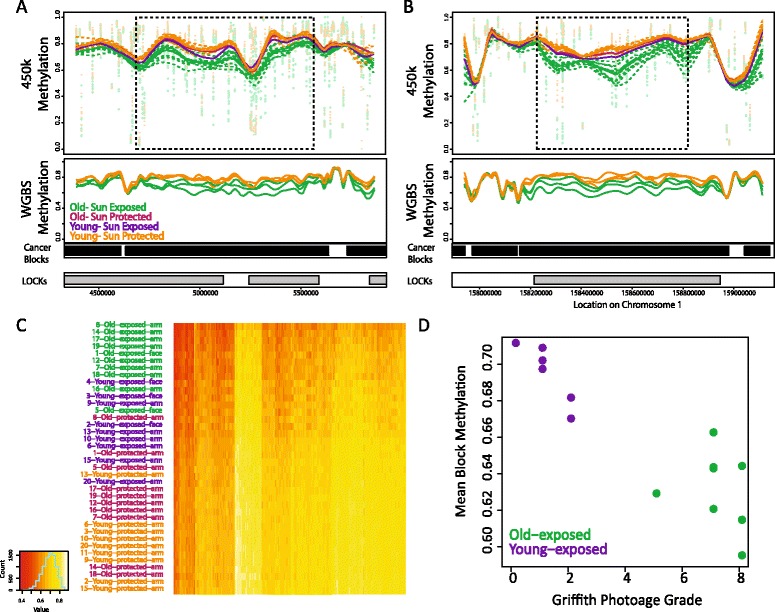
Block hypomethylation progresses from younger sun-protected to older sun-protected to younger sun-exposed to older sun-exposed tissue. **(A)** Example of a region identified as a block comparing older sun-exposed to younger sun-protected epidermal samples using 450k data. Top panel: methylation beta values (Methylated signal/Total signal) for 'collapsed' measurements of methylation from open sea probes in 450k data. These are methylation averages for each 1,500 bp open sea region calculated as part of the *minfi*’s 'block finder' algorithm. The points represent individual samples at each location, dotted lines show smoothed measurements across the region for each individual and solid lines represent the smoothed average for each group. The box demarcates the block identified using *Minfi*. Bottom panel: smoothed methylation beta values from WGBS data within the regions identified in 450k analysis. Horizontal bars indicate the locations of hypomethylated blocks identified previously in cancer and heterochromatin LOCKs [20]. **(B)** Example of a region identified as a block comparing older, sun-exposed and younger, sun-protected epidermal samples using 450k data, plotted as in (A). **(C)** Heatmap showing mean block methylation in all blocks identified comparing O-exp and Y-pro epidermis. Samples are ordered by mean methylation, and blocks are ordered by mean difference in methylation between O-exp and Y-pro samples. Red/yellow indicate lower/higher mean methylation levels, respectively. **(D)** Relationship between block methylation and Griffiths’ photoage grade. Mean methylation within all blocks identified comparing O-exp and Y-pro epidermis, versus Griffiths’ photoage grade assigned to the sample donor.

In previous studies comparing methylation in colorectal cancer and normal samples, large hypomethylated blocks spanning large, primarily CpG-poor regions of the genome were seen to be the major source of methylation change in cancer [20,21]. Given the large differences found by principal component analysis within epidermal samples, we hypothesized that similar large blocks might underlie the methylation changes seen with age and sun exposure. Recent advances in analysis of 450k data through the *Minfi* package now make it possible to identify such large blocks in addition to small DMRs by applying the Bumphunter approach to probes in open sea regions using a large smoothing window [24,27]. We began by using this block finder to identify any large blocks differentially methylated between the samples that appear most different upon principle component analysis, the epidermal O-exp and Y-pro samples. Sun exposed samples obtained from the face were included in this analysis. Identified blocks were filtered by size and the calculated family-wise error *P*-value. We identified 224 blocks, of which 223 were hypomethylated in the O-exp samples compared to Y-pro samples, with an average size of 443 kb (ranging from 202 kb to 1.3 Mb) and 9.2% reduction in DNA methylation, ranging from 5.2 to 16% (examples in Figure 2A,B; list of block locations in Additional file 3). These blocks cover a total of 99 Mb of the genome. It is likely that more blocks of hypomethylation are present in these samples but our detection is limited by the coverage of the 450k array in open sea regions.

We first sought to rule out confounding by the fact that exposed and protected samples are obtained from slightly different sites on the body (outer arm versus inner arm). If the large scale differences observed were due to body location *per se* and not chronic exposure, we should detect these changes comparing the exposed and protected samples obtained from younger individuals. To address this, we performed the block finding analysis comparing Y-exp and Y-pro samples. Using the same cutoffs applied previously, we identified only 12 regions that differ, encompassing 3.8 Mb of the genome - compared with 224 regions, encompassing 99 Mb for the O-exp versus Y-pro comparison (Additional file 4). While significant, the relatively small area involved (3.8 Mb compared to 99 Mb) indicates the magnitude of block hypomethylation observed comparing O-exp and Y-pro samples cannot be attributed only to body site differences. By contrast, when comparing the O-exp and O-pro samples, we identified hypomethylated blocks of a comparable magnitude to the O-exp and Y-pro comparison, 239 blocks encompassing 100 Mb, with a mean difference of 8.6% hypomethylated. While these analyses were performed without regard to individual specific changes, comparing the paired and unpaired t-statistics for each methylation location demonstrates that the unpaired analysis is conservative and only underestimates the magnitude of change present (Additional file 5). This further indicates that only long term sun exposure, as seen in older donors, is sufficient for hypomethylated blocks.

We also sought to determine if hypomethylation at specific regions may be linked to age alone by comparing O-pro and Y-pro samples. However, using the cutoffs applied previously, no blocks were detected. This is consistent with the lack of large scale change associated with intrinsic aging reported previously [16]. By contrast, when we compared O-exp and Y-exp samples, we identified 33 block regions with an average of 10% difference in methylation, 32 of which are hypomethylated with age, encompassing 12.7 Mb (Additional file 6). The presence of age-related hypomethylated blocks in the sun-exposed body site but not the sun-protected body site further demonstrates that hypomethylated blocks arise only in epidermal tissue affected by both aging and sun exposure. In contrast to the epidermis, all block finding comparisons in the data from dermal samples identified no significant blocks, consistent with the lack of differential clustering seen in the principal component analysis. Thus, hypomethylated blocks encompassing a significant portion of the genome are only observed in epidermal samples affected by both age and sun exposure. Results of all comparisons are summarized in Table 1.

**Table 1:** **Summary of blocks that vary with aging and sun exposure**

	**Number 450k**	**Total size 450k**	**Mean change 450k**	**Total size WGBS**	**Mean change WGBS**
**Epidermis**					
O-exp versus Y-pro (sun + age)	224	99 Mb	9.2%	670 Mb	9.0%
Y-exp versus Y-pro (sun/site)	12	3.8 Mb	9.3%	0.9 Mb	7.3%
O-exp versus O-pro	239	100 Mb	8.6%	1.5 Mb	7.5%
O-pro versus Y-pro (age)	0	-	-	6.2 Mb	7.1%
O-exp versus Y-exp	33	12.7 Mb	10.0%	8.5 Mb	8.0%
**Dermis**	**Number**	**Total width**	**Mean difference**		
O-exp versus Y-pro	0	-	-	NA	NA
Y-exp versus Y-pro	0	-	-	NA	NA
O-pro versus Y-pro	0	-	-	NA	NA
O-exp versus Y-exp	0	-	-	NA	NA

Hierarchical clustering of all 38 epidermal samples based on methylation within identified blocks demonstrated a progressive hypomethylation with age and sun exposure: the Y-pro samples are most methylated in blocks; O-pro and Y-exp samples have lower block methylation; and O-exp samples have the lowest methylation in identified blocks (Figure 2C). Furthermore, this showed individual variation in the magnitude of change in blocks, with some donors showing much larger changes than others. For example, donor 8 has the lowest methylation in the sun-exposed sample across most identified blocks, while the sun exposed sample from another older individual, donor 16, is more methylated within the identified blocks. Sun-exposed samples obtained from the face (denoted in Figure 2C) fall with sun-exposed samples obtained from the arm, indicating that the observed hypomethylation is not specific to the dorsal forearm.

### Confirmation of hypomethylated blocks by whole genome bisulfite sequencing

To confirm the presence of hypomethylated blocks related to age and sun exposure in epidermal tissue and to examine the whole genome, we obtained sun exposed and sun protected epidermal tissue from three additional younger donors (mean age 22 years, average Griffiths’ grade 0) and three additional older donors (mean age 77 years, average Griffiths’ grade 5) and performed WGBS. We generated sequencing data to a depth of 5.6× to 7.2× and analyzed it using the BSmooth algorithm, which was designed for analyzing low-coverage WGBS data and has been previously demonstrated to accurately estimate methylation levels at a single-base pair resolution by borrowing information from nearby CpGs [28]. After filtering reads with low quality measures, we obtained measurements for an average of 24,873,842 CpGs per sample (average of 88.15% coverage). Bisulfite conversion, assessed using spiked in lambda phage, ranged from 99.7 to 99.9% (details in Materials and methods; Additional files 7 and 8).

We first examined CpGs within the regions identified as blocks comparing the O-exp and Y-pro samples in 450k. All 450k O-exp/Y-pro blocks showed hypomethylation in the O-exp samples in the WGBS data, with 185 out of 224 blocks showing >5% mean hypomethylation in O-exp samples in the sequencing data, validating our finding of widespread hypomethylated blocks in O-exp epidermis. For block regions depicted in Figure 2A,B, methylation in WGBS samples is shown in the lower panel. Mean methylation difference from WGBS for all 450k blocks is provided in Additional file 3. When only methylation within blocks identified in 450k data was examined, we observed separate clustering of O-exp samples from Y-pro samples, indicating these regions are sufficient to differentiate groups within sequencing data (Additional file 9).

**Figure 3:**
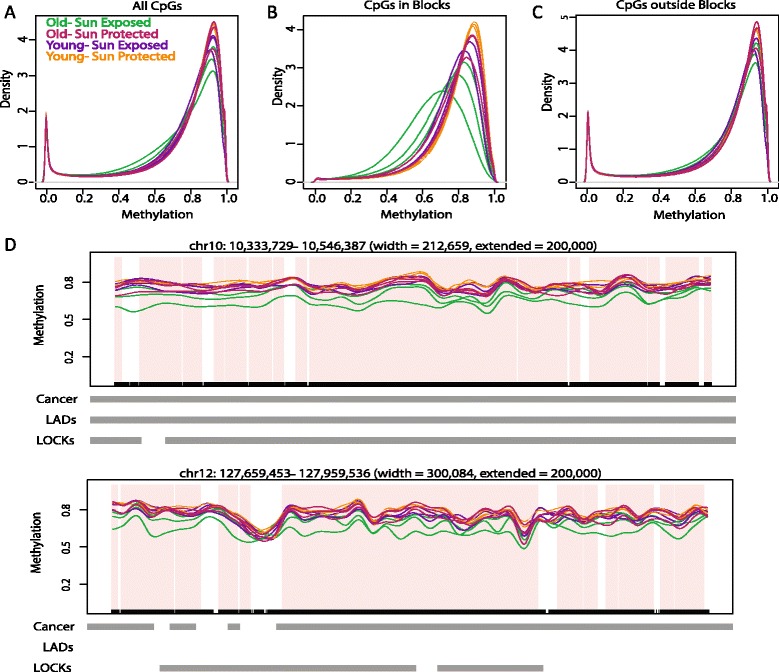
Hypomethylated blocks in O-exp samples replicated by whole genome bisulfite sequencing. **(A-C)** Hypomethylation is enriched in blocks. Distribution of high-frequency smoothed methylation values from CpGs with sufficient coverage from WGBS for (A) all CpGs, (B) CpGs in blocks, and (C) CpGs outside of blocks. **(D)** Examples of blocks identified by WGBS. Shown are smoothed methylation values within blocks identified comparing O-exp and Y-pro epidermis (pink).

Density plots of measured methylation at all CpGs with sufficient coverage in all sample groups showed hypomethylation in sun-exposed epidermal samples from older donors (Figure 3A). Using BSmooth [28], we identified blocks within our WGBS dataset. As in the 450k data, we identified large regions of change comparing the O-exp and Y-pro epidermal samples. The bisulfite sequencing analysis identified a similar mean loss in DNA methylation, 9% (ranging from 5 to 23%), but a much more substantial fraction of the genome than found by 450k analysis, that is, 670 Mb (21% of the genome). The mean loss in methylation comparing the more closely related groups found similar reductions in mean methylation but fewer numbers of blocks than in the 450k analysis, likely because of the smaller sample size for sequencing and thus reduced power to detect differences between the more closely related groups in the latter case (summarized in Table 1).

The observed global hypomethylation in O-exp samples is explained by CpGs within the identified blocks (Figure 3B,C). Thus, the results of this replication set independently confirmed our finding of hypomethylated blocks in samples affected by age and sun exposure. Examples of block regions identified in WGBS are shown in Figure 3D, and the complete locations of the hypomethylated blocks are listed in Additional file 10.

### Hypomethylated blocks overlap with heterochromatic domains

The increased genomic coverage provided by sequencing data allowed us to compare the identified regions with other genomic domains identified through sequencing-based methods. The hypomethylated O-exp versus Y-pro blocks in our data were seen to overlap strongly with previously reported hypomethylated blocks in human colon cancer [20] (odds ratio (OR) 8.5, *P* < 2 × 10^-16^). These O-exp versus Y-pro blocks similarly showed significant overlap with LOCKs (OR 3.4, *P* < 2 × 10^-16^) and with LADs (OR 3.5, *P* < 2 × 10^-16^) mapped in fibroblast cell lines [20,29]. Similar overlap of hypomethylated domains with these heterochromatin structures has been described in cancer and Epstein-Barr virus (EBV)-transformation of lymphocytes [20,30], suggesting a functional connection between the age and exposure associated alteration in large domains of DNA methylation and processes related to cancer.

In other studies, small age-related DMRs have been found to occur preferentially at regions marked by both H3K4me3 and H3K27me3 in embryonic stem cells (bivalent domains) [6,31]. We compared the identified blocks with regions identified as containing both H3K4me3 and H3K27me3 in human embryonic stem cells [32] and saw no enrichment (OR 0.68, *P* < 2 × 10^-16^). Further, we note that only 17 out of 353 of the CpGs seen to correlate strongly with chronological age across multiple tissues by Horvath [33] are found within the identified block regions. This suggests that the observed large regions of hypomethylation in aged, sun-exposed epidermis represent a distinct change from the chronological age-specific changes reported across other studies.

### Methylation in blocks associated with clinical measures

Given the observed individual variation, we sought to determine how well block methylation levels predict clinical changes associated with skin aging. For each skin donor, a dermatologist evaluated their degree of apparent skin aging in sun-exposed and sun-protected regions using two established scales. Griffiths’ photodamage age grading measures coarse and fine wrinkling, photopigmentation and yellowing [34], and showed a significant correlation with mean block methylation in sun-exposed epidermal samples (R^2^ = 0.61, *P* < 0.001; Figure S4A in Additional file 11). Much of the variation in this relationship appears to be linked to the younger, sun-exposed samples obtained from the face, which appear to be more hypomethylated for a given age grade than the arm samples. When these samples are removed from regression, correlation is even stronger, with an R^2^ = 0.81, *P*-value <0.001 (Figure 2D). Helfrich’s photo-protected skin aging scale, in contrast, was developed for sun-protected skin and measures more nuanced fine wrinkling [35]. This sun-protected scale showed far less pronounced correlation with block methylation in sun-protected samples (R^2^ = 0.16, *P* = 0.1; Figure S4B in Additional file 11), suggesting that the block hypomethylation correlates with clinical grading primarily in epidermal samples affected by sun exposure. Thus, methylation levels in O-exp versus Y-pro blocks decrease with chronic exposure, varying with the degree of clinically appreciable photoaging.

To explore how genetic diversity may relate to the observed hypomethylated blocks in aged, sun-exposed skin, we examined signal at 65 single nucleotide polymorphism (SNP) probes included on the 450k array. None of the 65 probes showed a significant difference (adjusted *P*-value <0.05) between samples from older and younger individuals (*P*-values for all probes in Additional file 12), even though these probes were chosen to discriminate genetic structure [23]. Additionally, we compared identified block regions to the genetically controlled methylation clusters (GeMes) mapped in a recent work using data from two large Caucasian cohorts [36], and genetically controlled CpGs identified using a large cohort of female twins through the MuTHER study [37]. Of the identified O-exp versus Y-pro blocks, 84 contain GeMes, although the GeMes account for only 6.6 Mb of the identified 99 Mb, indicating that a large area is involved in which methylation is not directly linked to genotype. Similarly, of the 13,378 probes involved in our identified O-exp versus Y-pro blocks, only 3,236 were linked to genotype. GeMes within the identified blocks are denoted in Additional file 3. Furthermore, if the effect observed were genetically driven, hypomethylated blocks would be present in both samples obtained from the involved individuals, but they are found only in older, sun-exposed samples.

Given previously reported relationships between DNA methylation and body mass index (BMI) and smoking [37-39], we compared methylation levels within the identified blocks to BMI and between smokers and non-smokers. We observed no significant relationship between BMI and block methylation (R^2^ = 0.02, *P* = 0.56) or smoking status for donors for which this information was available (R^2^ = 0.20, *P* = 0.32) (Figure S5A,B in Additional file 13).

### Differential gene expression with epidermal aging and sun exposure

To determine the relationship between hypomethylated blocks observed in epidermal samples with sun exposure and aging, we obtained RNA from epidermal samples from a subset of the donors, including three Y-pro epidermal samples and four O-exp epidermal samples (individual donors used denoted in Additional file 1). Expression data were obtained using Affymetrix arrays. When we compared O-exp and Y-pro samples using gene set enrichment analysis (GSEA) [40], 88 pathways were identified as significantly enriched based on a family wise error rate (FWER) <0.05. Intriguingly, this analysis identified three pathways associated with UV exposure *in vitro*, as well as multiple pathways linked to cell cycle and proliferation (full results in Additional file 14). To understand the relationship between sample groups, we used *limma* to compare each group of samples. When comparing the O-exp and Y-pro samples, we observed more probes with upregulation in the O-exp samples (Figure S6A in Additional file 15). When we considered the intrinsic aging comparison (O-pro versus Y-pro), we did not observe this trend (Figure S6C in Additional file 15). When we considered the Y-exp versus Y-pro comparison, we noted a similar trend towards upregulation in the sun-exposed samples (Figure S6E in Additional file 15). This indicates that, unlike the pattern observed with methylation, the global changes in gene expression are driven by the exposure/body site differences, not the combination of age and sun exposure.

Given our observation of widespread methylation changes within the epidermal samples, we sought to use our gene expression data to confirm that no large shifts in cell type composition may be confounding our analysis. The second most common cell type in the epidermis, melanocytes (approximately 3% of cells), can be distinguished from keratinocytes based on the presence of MITF and SOX10 [41]. To determine if large changes in melanocyte levels are present in our samples, we examined expression probes measuring expression of these markers (three measuring MITF and two measuring SOX10). None of the examined probes showed a significant difference in expression comparing O-exp and Y-pro samples, and none showed a log fold change greater than 0.5 (Additional file 16).

In order to determine how patterns of expression relate to the identified hypomethylated blocks, data were normalized using fRMA and probes were classified as expressed or unexpressed using gene expression barcode [42]. When expression data were compared to the locations of photoaging blocks, probes within blocks were less likely to be expressed than those outside (OR 0.38, *P* < 0.001), consistent with the observed overlap between blocks and heterochromatic regions.

### Blocks hypomethylated with sun exposure and aging are hypomethylated in squamous cell carcinoma

**Figure 4:**
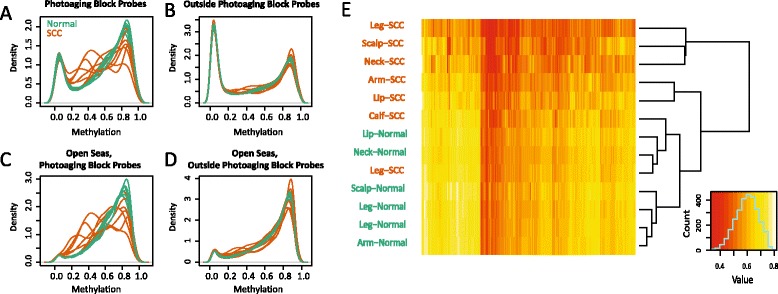
Blocks identified in photoaged skin are more hypomethylated in squamous cell cancer (SCC). **(A)** Distribution of methylation values in 450k probes measuring CpGs within the identified age- and sun exposure-associated blocks. **(B)** Distribution of methylation values in 450k probes measuring CpGs outside of the identified age- and sun exposure-associated blocks. **(C)** Distribution of methylation values in 450k probes measuring CpGs within open sea regions and within the identified age- and sun exposure-associated blocks. **(D)** Distribution of methylation values in 450k probes measuring CpGs within open sea regions and outside the identified age- and sun exposure-associated blocks. **(E)** Heatmap showing mean methylation in blocks identified comparing O-exp and Y-pro epidermis. Samples and blocks are ordered by hierarchal clustering. Yellow/red indicate higher/lower methylation, respectively.

Having observed an overlap between our chronic exposure blocks and hypomethylated domains observed in colon cancer, we sought to determine the relevance of our identified domains to a cancer that develops in epidermis with chronic sun exposure, squamous cell carcinoma (SCC). We obtained seven SCC tissue samples and six normal skin samples from the same body sites as the SCC samples and analyzed DNA methylation using the 450k array. Strikingly, we observed hypomethylation of SCC samples compared with normal samples when examining probes within the identified chronic exposure blocks (Figure 4A), which is not seen when examining probes outside of these regions (Figure 4B). This difference is seen even when examination is limited to probes within the constitutively methylated open sea probes (Figure 4C,D). For 221 out of 223 identified hypomethylated blocks, SCC samples had lower mean methylation than normal samples (difference in mean methylation noted in Additional file 3). Clustering of these data based on mean methylation within identified photoaging blocks distinguishes most SCC from normal samples (Figure 4E).

### Methylation age correlates strongly with chronological age

In a recent work, Horvath [33] built an age-prediction model using methylation at 353 cytosines probed on the 450k array which allows calculation of a 'methylation age' for any given sample. Horvath reports these methylation ages are highly correlated with chronological age using a wide range of tissue samples. We applied Horvath’s model to calculate methylation age for our samples to determine how the widespread hypomethylation we observe in O-exp epidermis relates to methylation age. We observed a strong correlation between chronological age and methylation age for both dermis (R^2^ = 0.91, *P* < 2.2e-16) and epidermis (R^2^ = 0.83, *P* < 1.1 e-15) (Figure S8A,B in Additional file 17).

### Small differentially methylated regions overlap with polycomb targets

We next used our 450k data to identify small DMRs. We used the method of 'bump hunting', which identifies genomic regions in which methylation levels at consecutive measured locations are associated with the outcome of interest. That method was initially developed for the CHARM array-based methylation method but was adapted for the Minfi package for the 450k array [24,27]. Significance testing was performed by permutation analysis. Comparing O-exp and Y-pro epidermal samples, we identified 166 small DMRs with at least 10% difference in methylation and an adjusted family-wise error rate <0.1. These included 90 hypermethylated and 76 hypomethylated DMRs (listed in Additional file 18). The average size of these DMRs was 460 bp, and the average change in methylation was 37% (ranging from 23 to 64%), indicative of local regional effect on DNA methylation, similar to what is seen in tissue and cancer DMRs.

As we considered with the block regions, we asked what changes were specific to age or sun exposure. Similarly to the block analysis, we identified a considerably smaller number of DMRs specific to sun exposure (30 significant DMRs found comparing Y-exp and Y-pro samples versus 166 comparing O-exp and Y-pro) and specific to age (2 significant DMRs found comparing O-pro and Y-pro samples versus 166 comparing O-exp and Y-pro). Within dermal samples, we again identified fewer regions of change than in the epidermis: nine significant O-exp and Y-pro DMRs were found, with even fewer sun-specific and age-specific DMRs. Complete DMR counts from all comparisons are shown in Additional file 19. These results suggest that small DMRs, like blocks, require both age and sun exposure and occur predominantly in the epidermis, although the cumulative size of these DMRs was less than 1% of the area covered by large blocks on 450k.

In other studies, small age-related DMRs have been found to occur preferentially at regions marked by repressive chromatin, bivalent domains, and targets of Polycomb repressive complex 2 [6,31,43,44]. To determine if this overlap is found in the identified small DMRs, we compared the identified regions with regions marked by H3K4me3 and H3K27me3 in normal human epidermal keratinocytes (NHEK cells) as identified by the ENCODE project [45]. To control for the background enrichment from the 450k array, we determined the significance of identified overlaps by generating length- and probe number-matched random regions from all array probes. We called an overlap significant if there was more overlap between identified DMRs and chromatin mark than >95% of randomly generated region sets. We observed a significant overlap between hypermethylated O-exp and Y-pro DMRs and regions marked by H3K27me3 in NHEK cells, but not regions marked by H3K4me3. Hypomethylated OE-YP DMRs did not overlap significantly with either mark.

## Discussion

A comprehensive analysis of DNA methylation in aging and sun-exposed skin reveals widespread DNA hypomethylation of large blocks that overlap heterochromatin domains and nuclear lamin-associated domains. The greatest difference was between older, sun-exposed and younger, sun-protected epidermal samples, indicating that both age and sun exposure status is necessary for widespread hypomethylation. We further observe these same regions to account for the majority of the hypomethylation in SCC samples, suggesting a possible link between widespread hypomethylation in cancer and the changes observed in normal epidermis.

The most unexpected result of our analysis was the genome-scale differential methylation seen in epidermal samples affected by both age and exposure, as evidenced by the distinct clustering in principal component analysis. While most population studies of aging and environmental exposures have focused on methylation changes in single CpGs or small DMRs [4,6,33], here we identify changes across large block regions encompassing approximately 20% of the genome. This extent of altered methylation suggests that the change associated with chronic environmental stress should be studied on a genome scale, rather than only focusing on changes in DNA methylation in discrete regions. While our sample size is relatively small, the large scale change identified is informative for future study. The overlap of our identified blocks with other structural domains, including LADs and LOCKs, further supports the idea that the observed changes in DNA methylation are indicative of alteration to the epigenomic structure. Future studies of chronic exposure should examine methylation at a similar, genome scale to determine if the changes identified in our sample set are more broadly relevant.

We chose to examine skin in this study because it offers a relatively homogenous system with regard to cell type for studying aging and exposure. Once separated, the epidermis in particular consists of 90 to 95% keratinocytes in both sun-exposed and sun-protected body sites [11]. The other main cell types in the epidermis, melanocytes and Langerhans cells, account for a small percentage of cells - there are approximately 36 keratinocytes per melanocyte and approximately 53 keratinocytes per Langerhans cell [46]. While small increases in melanocyte density in sun-exposed body sites and small decreases in melanocyte density with age have been reported, these are not of the correct pattern or sufficient magnitude to confound the observed blocks of hypomethylation: comparing sun-exposed and sun-protected body sites from young and old individuals shows up to a twofold increase in melanocytes in heavily sun-exposed sites and a decrease in melanocyte density by 6 to 8% per decade in both sun-exposed and sun-protected locations [47]. As melanocytes account for approximately 3% of cells present, a doubling or complete loss of these cells could account for up to a 3% change in methylation, smaller than the magnitude of change observed in blocks. Further, as we demonstrate, the observed hypomethylated blocks are not present comparing sun-exposed and sun-protected body sites in young individuals, so they cannot be attributed to the increased percentage of melanocytes present in sun-exposed body sites of both young and old individuals. The age related decrease in melanoctyes would amount to approximately a 1% change in cell composition over four decades (32% decrease in 3% of cells), which is again not sufficient to account for the changes we observe. Consistent with the very small changes in cell composition reported in the literature, analysis of gene expression data from our samples indicates no significant changes in melanocyte marker genes.

In the recent literature, multiple methods have been developed to *in silico* correct for cell type heterogeneity, including SVA, EWASher and refFreeEwas [48-50]. All of these methods assume that the signal between cases and controls is small compared to the effect of cell type heterogeneity and that any large scale change identified in principle component analysis is due to cell composition changes. Using these methods will therefore remove any large scale signal between cases and controls, even if these differences are not due to cell composition effects. An example is given by the EWASher paper in which applying the methodology to comparison of colon cancer tumors and normal colon leaves only two CpGs identified as significantly differentially methylated in cancer, contradicting 25 years of literature on this disease [49], and acknowledged by the authors themselves to be a significant blind spot of such correction methods [51]. Despite the significant body of evidence supporting the absence of large scale cell composition changes in our samples, the methylation changes we describe encompass a large portion of the genome and are large enough to affect clustering (Figure 1), so it is not surprising that the application of these algorithms to our data removes the widespread hypomethylation observed in the older, sun-exposed samples. However, given the substantial evidence indicating no large changes in cell composition with aging and sun exposure, we attribute this to the degree of differential methylation within the keratinocyte population and not changes in cell type composition.

The identified blocks are distinct from the single CpGs used to calculate 'methylation age' by Horvath and the small hypermethylated DMRs overlapping bivalent chromatin domains identified as markers of chronological age in other studies [6,31,33]. We see no evidence of hypomethylation within our blocks or within 450k open sea probes associated with chronological aging in publically available data from adipose tissue or peripheral blood [4,36] (Figure S9A-D in Additional file 20), reinforcing our finding that block hypomethylation in epidermal tissue occurs only with chronic exposure, not aging alone. Heyn *et al*. report widespread hypomethylation with age in CD4+ T cells from WGBS of cells sorted from one newborn and one centenarian [5], indicating that large blocks of hypomethylation may be relevant in other models of aging, although we see only a moderate overlap of our identified blocks with hypomethylated CpGs in their study (OR = 1.77; Figure S9E,F in Additional file 20) and the use of single samples in that study makes it difficult to draw further conclusions about the regions involved.

A functional role of altered block methylation is supported by the correlations between block methylation and clinical measures of skin aging. Our analysis of the relationship between hypomethylated blocks and gene expression is limited by the low number of samples available for gene expression analysis. In studies of cancer and EBV transformation, large publically available expression datasets have allowed identification of a clear relationship between hypomethylation and variability of expression of genes within blocks [20,30]. Another recent study linked loss of methylation in gene-poor regions with activation of distal enhancers, indicating that loss of methylation may have functional consequences beyond regulation of nearest genes [52]. Consistent with an environmental exposure-driven effect, the magnitude of age- and sun-related change was greater in epidermal samples compared with dermal samples. These results suggest that molecular aging and UV exposure studies focused on dermis or full thickness biopsy may miss the relevant epigenetic effects [53].

The occurrence of blocks of hypomethylation related to age and sun exposure in regions previously seen to be hypomethylated in cancer is highly intriguing. Similar blocks identified in colon cancer samples were seen to develop very early in cancer progression [20], suggesting epigenetic reorganization may be an early event in carcinogenesis. While the blocks observed in our study encompass a smaller portion of the genome and have a smaller magnitude of change, their occurrence in non-malignant tissue suggests that epigenomic change may be initiated by exposures, rather than only during oncogenesis. Our observation in this work, that blocks identified as hypomethylated with chronic exposure in non-malignant tissue are hypomethylated in SCC, while most other sites are not, suggests that these changes may occur prior to malignancy. Our identification of this subset of the 'cancer blocks' that become hypomethylated with chronic exposure offers a potential target for chemoprevention strategies.

We note that these results suggest that an early change quantitatively related to chronic exposure in skin is a large-scale epigenomic alteration that was missed by earlier studies focused on individual genes or loci such as CpG islands. Intriguingly, a recent study by Cruickshanks *et al*. identifies similar large regions of hypomethylation in cells cultured to replicative senescence [54], linking such loss of methylation to cell turnover and cell stress in culture. Further, Takeuchi *et al*. [55] recently demonstrated that repeated exposure of aged cells to UVA induces expression of progerin, the mutated form of Lamin A seen in Hutchinson-Guilford progeria, and abnormal nuclear morphology. This is intriguing given the observed overlap between our blocks and lamina-associated domains. While speculative, a general model consistent with these data is that repeated stress and cell turnover leads to progressive destabilization of heterochromatin along the nuclear membrane, with attendant changes in DNA methylation. These changes could serve both as a molecular epigenetic clock of environmental exposure and a potential target of increased risk. They fit well an age-associated epigenetic drift hypothesis [9]. These results may have therapeutic implications. For example, future experiments should be performed to determine whether modifiers of sun damage, such as retinoic acid, or specific inhibitors of laminopathies, such as lonafarnib [56], might modify the development of the epigenomic alterations described here. Finally, we emphasize that the changes observed here may be restricted to the specific sample types and method of analysis used here.

## Conclusions

Our data demonstrate widespread blocks of hypomethylation, involving 670 Mb of the genome associated with chronic exposure in older, sun-exposed epidermal samples. The degree of hypomethylation correlates with clinical measures of photo-aging. We observe these same reasons to be similarly hypomethylated in SCC. These data implicate large scale epigenomic change in mediating the effects of environmental damage with photo-aging.

## Materials and methods

### Human subjects and tissue samples

Twenty-six healthy volunteers took part in the study. Samples from 20 of these donors were used for 450k analysis, and samples from the additional six donors were used for WGBS. All participants provided written informed consent, and this study was approved by the Johns Hopkins Medicine Institutional Review Board (IRB# NA_00041408 for sun-exposed and sun-protected samples, NA_0036868 for SCC samples). All human experimentation was performed in accordance with the Declaration of Helsinki. Exclusion criteria included use of topical medications within 2 weeks of sampling, active or dormant skin conditions, and pregnancy. To control for pigmentation, only Caucasian subjects were selected. For each subject, a dermatologist evaluated the degree of apparent skin aging using two established scales: Griffiths’ photodamage age grading measures coarse and fine wrinkling, photopigmentation and yellowing [34] while Helfrich’s photo-protected skin aging scale, in contrast, was developed for sun-protected skin and measures more nuanced fine wrinkling [35]. Paired punch biopsy samples, 4 mm in diameter, were collected under local anesthesia from the outer forearm or lateral epicanthus (sun-exposed area) and upper inner arm (sun-protected area). Immediately after removal, samples were washed in phosphate-buffered saline and transferred to DMEM (Gibco, Grand Island, NY, USA) containing dispase (2 U/ml). Following dispase treatment overnight at 4°C, epidermis and dermis were separated, flash frozen and stored at -140°C. SCC containing tissue was obtained from 10 previously diagnosed patients (8 females and 2 males; median age 67 years) undergoing either Mohs micrographic or excisional surgery for treatment of their skin cancer. During repair of the surgical defect, standing cone deformity 'dog ear' tissue was retained and submitted as normal control tissue. Samples were OCT embedded, frozen and stored at -80°C.

### Histology

Dispase-separated, flash frozen epidermal sections were cut into 5 μm sections and stained with hematoxylin and eosin. Sections were imaged using a Vectra Imaging system.

### DNA isolation

DNA was isolated from biopsy samples (epidermis and dermis) and SCC samples using the MasterPure DNA Purification Kit (Epicentre, Madison, WI, USA) according the manufacturer’s protocol.

### 450k array

DNA was quantified using Quant-iT Picogreen Reagent (Invitrogen, Grand Island, NY, USA) according to the manufacturer’s instructions. DNA (1 μg) was bisulfite treated using the EZ DNA methylation kit (Zymo Research, Irvine, CA, USA) according to the manufacturer’s specifications for the 450k array. Converted genomic DNA was eluted in 22 μl of elution buffer. DNA methylation level was measured using Illumina Infinium HD Methylation Assay (Illumina) according to the manufacturer’s instructions.

### 450k analysis

All analyses were performed using R 3.0.3. Raw intensity files were obtained and processed using the Minfi package to obtain methylation ratios (beta values). Samples were normalized using the Illumina preprocessing method implemented in Minfi. We applied multiple quality control measures to remove questionable arrays or probes. We examined 450k array control probes to assess many measures of assay efficiency and calculated median methylated and unmethylated measurements for each sample. We removed probes that had an annotated SNP (dbSNP137) at the single base extension or CpG site (17,541 probes removed).

To identify blocks, we used the block finder as described elsewhere [24]. Briefly, we applied the Bumphunter approach to the open sea probes using a large smoothing window. The estimates were thresholded based on a 5% difference in methylation beta values. Blocks were filtered to include only those >200 kb. Significance was assigned based on permutation testing; a cutoff of adjusted *P*-value (family-wise error rate) <0.05 was used.

To identify small DMRs, we used the bump hunting technique as previously described [57]. Estimated differences were controlled for sex and body site (face or arm) in the linear model. The estimates were thresholded using a 0.1 difference in beta values (approximately 10% difference in methylation). Significance was assigned based on permutation testing; a cutoff of adjusted *P*-value (family-wise error rate) <0.1 was used.

To examine overlap with histone marks, we downloaded ChIP-Seq peaks from NHEK cells (Lonza CC-2501) generated by the Bernstein-Broad group for the ENCODE project (Gene Expression Omnibus (GEO) accession GSM733701, GSM733720) and determined how many of our identified DMRs overlap with each set of marks. To assess the significance of overlap with the identified DMRs, we generated random regions from 450k probes with the same width and probe number as the identified regions, determined how many of these random regions overlap with each histone mark and repeated this procedure 1,000 times. The DMR list was considered to significantly overlap with a histone mark if it contained more overlaps than >95% of randomly generated lists.

### Affymetrix microarray expression analysis

RNA was isolated from epidermal samples using Trizol Reagent (Life Technologies, Grand Island, NY, USA) according to the manufacturer’s instructions followed by clean up using the RNeasy kit (Qiagen, Valencia, CA, USA) following the RNA cleanup protocol. Genome-wide gene expression analysis was done using Affymetrix U133 Plus 2.0 microarrays according to Affymetrix’s specifications. Data were normalized using fRMA as previously described [58] and expression was determined using gene expression barcode [41]. Probes were classified as expressed if the mean expression Z-score in at least one group was >2.54. Differential expression was determined using limma [59]. GSEA was performed using the gene pattern suite GSEA module [60]. Significance was assessed using 1,000 permutations of gene sets.

### Whole genome bisulfite sequencing libraries

Bisulfite sequencing libraries were constructed using the Illumina TruSeq DNA Library Preparation kit protocol with the following modifications. Unmethylated lambda DNA (10 ng) was added to 1 μg of genomic DNA prior to shearing in order to monitor bisulfite conversion efficiency. After shearing, end repair was performed using a modified protocol to prevent introduction of non-genomic cytosines by using only dATP, dGTP and dTTP nucleotides with a mixture of Klenow DNA polymerase, T4 DNA polymerase and T4 polynucleotide kinase. After purification, samples were bisulfite converted and purified using Zymo EZ DNA Methylation Gold. Bisulfite converted libraries were amplified using a mixture of uracil-insensitive polymerases, Denville Choice Taq and Agilent Pfu. Samples were amplified for 10 cycles of PCR.

### Whole genome bisulfite sequencing analysis

All analyses were performed using R 3.0.1. To process sequencing data, we ran the BSmooth [28] bisulfite alignment pipeline (version 0.4.5-beta) on the 100-by-100 bp HiSeq 2000 paired-end sequencing reads obtained for each sample, using Bowtie2 version 2.0.1 [61] and the hg19 build on the human genome as well as the genome for lambda phage. Additional file 7 summarizes the alignment results. After alignment, BSmooth was used to extract read-level measurements, summarized in Additional file 8. We filtered out measurements with mapping quality <20 or nucleotide base quality <10 and we removed measurements from the 5′ most 10 nucleotides of both mates. BSmooth was used to sort read-level measurements by genomic coordinates and compile a summary table.

Next, BSmooth was used to identify large hypomethylated blocks as described in detail previously [20,28,30]. CpGs with coverage of 2 or greater in each sample group (O-exp, O-pro, Y-exp, Y-pro) were included in the analysis. We used the same cutoffs used in studies of cancer, specifically a t-statistic cutoff of -2, 2. We estimated variance based on the younger, sun-protected samples. Identified blocks were filtered to include only blocks >10 kb and with a mean difference of >5%.

### Data availability

Array and sequencing data are available in GEO under accession number GSE52980.

## Additional files

Additional file 1: Table S1. Demographics of skin donors used in array and sequencing analysis. Additional file 2: Figure S1. Hemotoxylin and eosin staining of dispase-separated epidermal punches. Additional file 3: Table S2. Locations and characteristics of the O-exp versus Y-pro blocks identified via 450k analysis. Additional file 4: Table S3. Locations of the Y-exp versus Y-pro blocks identified via 450k analysis. Additional file 5: Figure S2. t-Statistics for the O-exp versus O-pro comparison calculated using paired versus unpaired tests. Additional file 6: Table S4. Locations of the O-exp versus Y-exp blocks identified via 450k analysis. Additional file 7: Table S5. Alignment results for sequencing. Additional file 8: Table S6. Sequencing coverage. Additional file 9: Figure S3. Clustering of WGBS samples using methylation within the O-exp versus Y-pro blocks identified in 450k analysis. Additional file 10: Table S7. Locations of O-exp versus Y-pro blocks identified using WGBS analysis. Additional file 11: Figure S4. Mean methylation within blocks identified comparing O-exp and Y-pro epidermis versus Griffiths’ photo age grade for all sun-exposed epidermal samples and mean block methylation versus Helfrich’s photo-protected age grade for all sun-protected epidermal samples. Additional file 12: Table S8. SNP probes within the 450k array and a *P*-value testing their association with young versus old donors. Additional file 13: Figure S5. Mean methylation within blocks identified comparing O-exp and Y-pro epidermis versus BMI and smoking status. Additional file 14: Table S9. Gene sets identified as significantly enriched in GSEA. Additional file 15: Figure S6. Volcano plots and the distribution of *P*-values for differential expression for the O-exp versus Y-pro, O-pro versus Y-pro and Y-exp versus Y-pro comparisons. Additional file 16: Figure S7. Expression levels of melanocyte-specific markers in our sample group. Additional file 17: Figure S8. Correlation between the methylation age calculated with Horvath’s algorithm and chronological age in dermal and epidermal samples. Additional file 18: Table S10. Locations of identified O-exp versus Y-pro small DMRs identified via 450k analysis. Additional file 19: Table S11. Results of small DMR finding in 450k data. Additional file 20: Figure S9. Demonstrates no age-related methylation changes within the blocks identified comparing O-exp and Y-pro epidermis in three public methylation data sets from peripheral blood and adipose tissue.

## Abbreviations

BMI: body mass index
DMR: differentially methylated region
EBV: Epstein-Barr virus
GeMe: genetically controlled methylation cluster
GEO: Gene Expression Omnibus
GSEA: gene set enrichment analysis
LAD: lamin-associated domain
LOCK: large organized chromatin lysine-modification
NHEK: normal human epidermal keratinocyte
OR: odds ratio
PCR: polymerase chain reaction
SCC: squamous cell carcinoma
SNP: single nucleotide polymorphism
UV: ultraviolet
WGBS: whole genome bisulfite sequencing
