# Supplementary figures and images for: Age and sun exposure-related widespread genomic blocks of hypomethylation in nonmalignant skin
Source: Genome Biol. 2015 Apr 16;16(1):80. doi: 10.1186/s13059-015-0644-y (PMC4423110; doi:10.1186/s13059-015-0644-y)

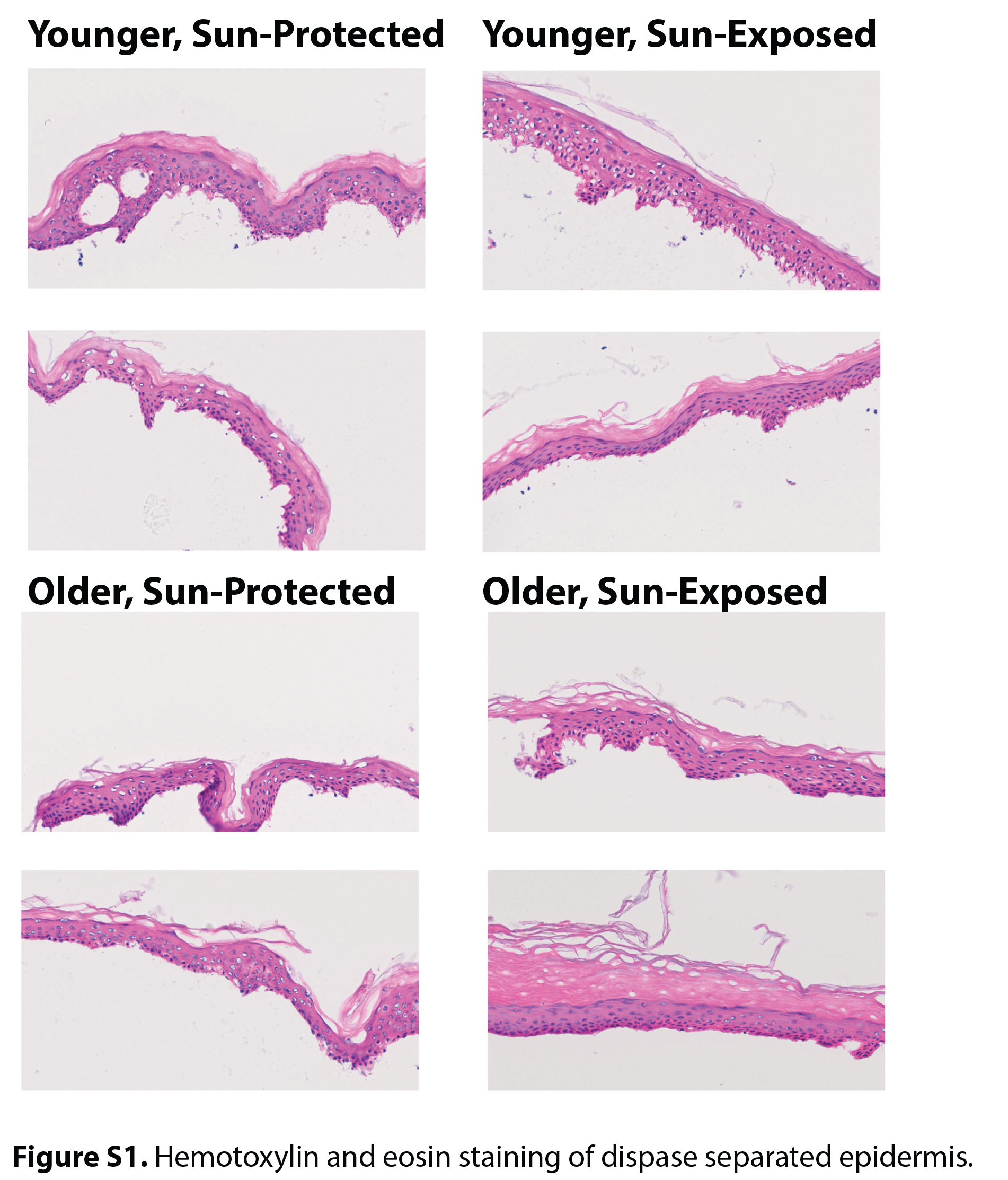

Supplement: Additional file 2: Figure S1. — Hemotoxylin and eosin staining of dispase-separated epidermal punches. [file 13059_2015_644_MOESM2_ESM.jpeg]
