# Supplementary material for: Age and sun exposure-related widespread genomic blocks of hypomethylation in nonmalignant skin
Source: Genome Biol. 2015 Apr 16;16(1):80. doi: 10.1186/s13059-015-0644-y (PMC4423110; doi:10.1186/s13059-015-0644-y)
Supplement: Additional file 5: Figure S2. — t-Statistics for the O-exp versus O-pro comparison calculated using paired versus unpaired tests. [file 13059_2015_644_MOESM5_ESM.pdf]

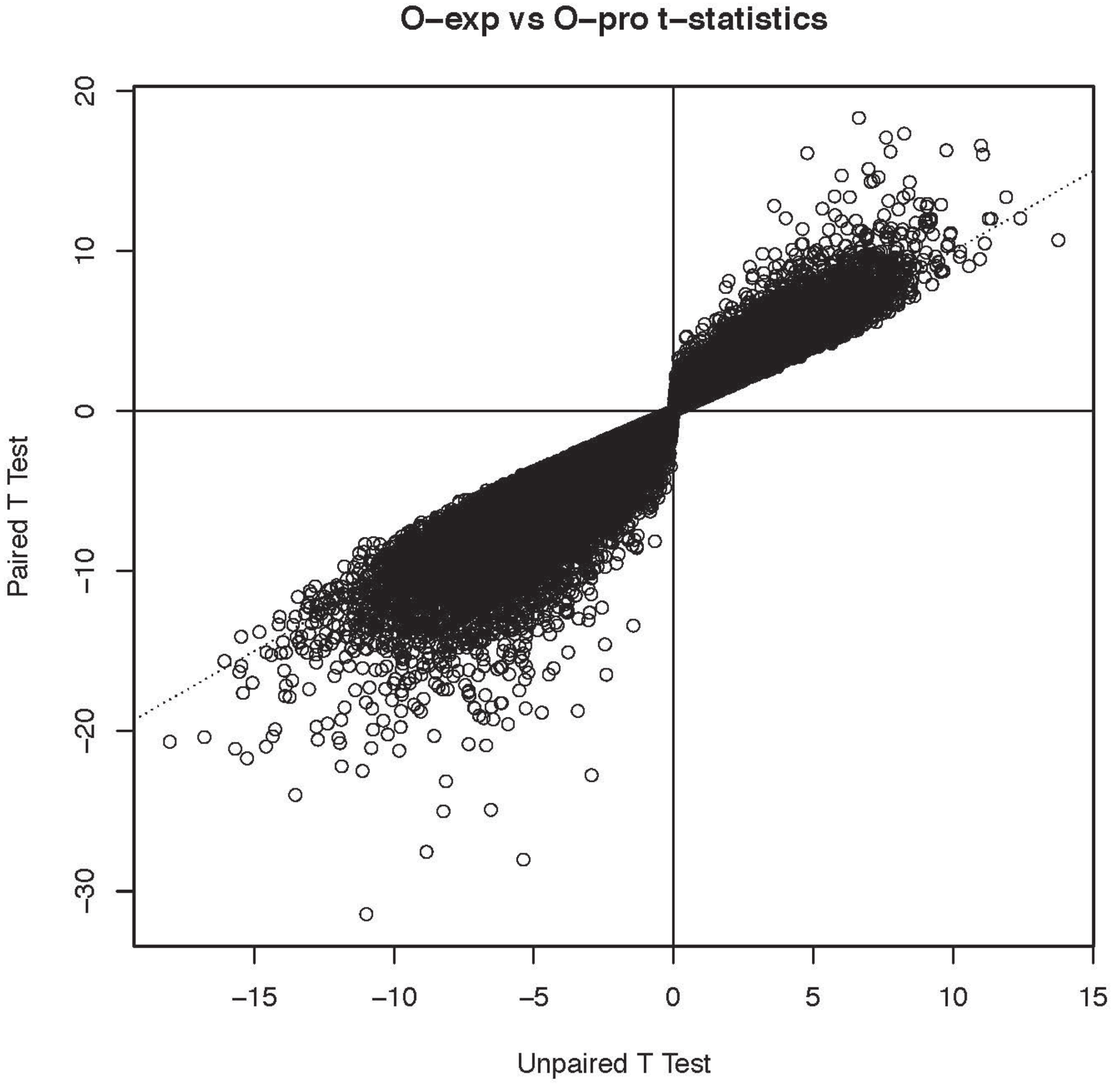

Figure S2. The unpaired analysis is consverative relative to a paired test. Shown are the t-statistics calculated for each probe comparing sun-exposed and sun-protected samples from older individuals using a paired t-test versus those calculated using an unpaired t-test.
