# Supplementary material for: Age and sun exposure-related widespread genomic blocks of hypomethylation in nonmalignant skin
Source: Genome Biol. 2015 Apr 16;16(1):80. doi: 10.1186/s13059-015-0644-y (PMC4423110; doi:10.1186/s13059-015-0644-y)
Supplement: Additional file 9: Figure S3. — Clustering of WGBS samples using methylation within the O-exp versus Y-pro blocks identified in 450k analysis. [file 13059_2015_644_MOESM9_ESM.pdf]

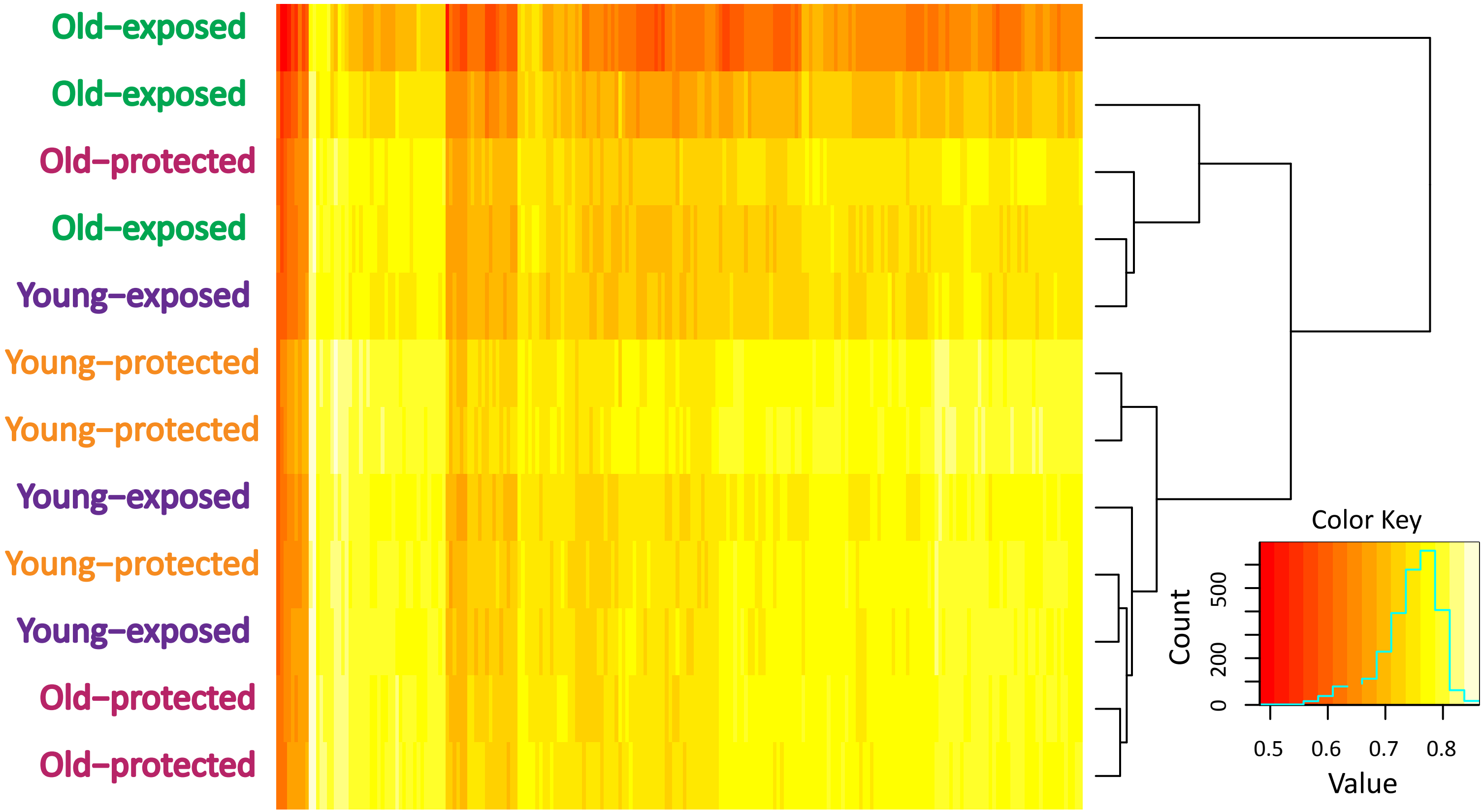


**Figure S3.** Hypomethylated blocks identified in 450k data separate O-exp and Y-pro samples  analyzed using WGBS.  Heatmap showing mean methylation from WGBS data in regions identified as blocks i comparing O-exp and Y-pro epidermis in 450k Data. Samples and blocks are ordered by hierarchal clustering. Yellow/red indicate higher/lower methylation, respectively.
