# Supplementary material for: Age and sun exposure-related widespread genomic blocks of hypomethylation in nonmalignant skin
Source: Genome Biol. 2015 Apr 16;16(1):80. doi: 10.1186/s13059-015-0644-y (PMC4423110; doi:10.1186/s13059-015-0644-y)
Supplement: Additional file 11: Figure S4. — Mean methylation within blocks identified comparing O-exp and Y-pro epidermis versus Griffiths’ photo age grade for all sun-exposed epidermal samples and mean block methylation versus Helfrich’s photo-protected age grade for all sun-protected epidermal samples. [file 13059_2015_644_MOESM11_ESM.pdf]

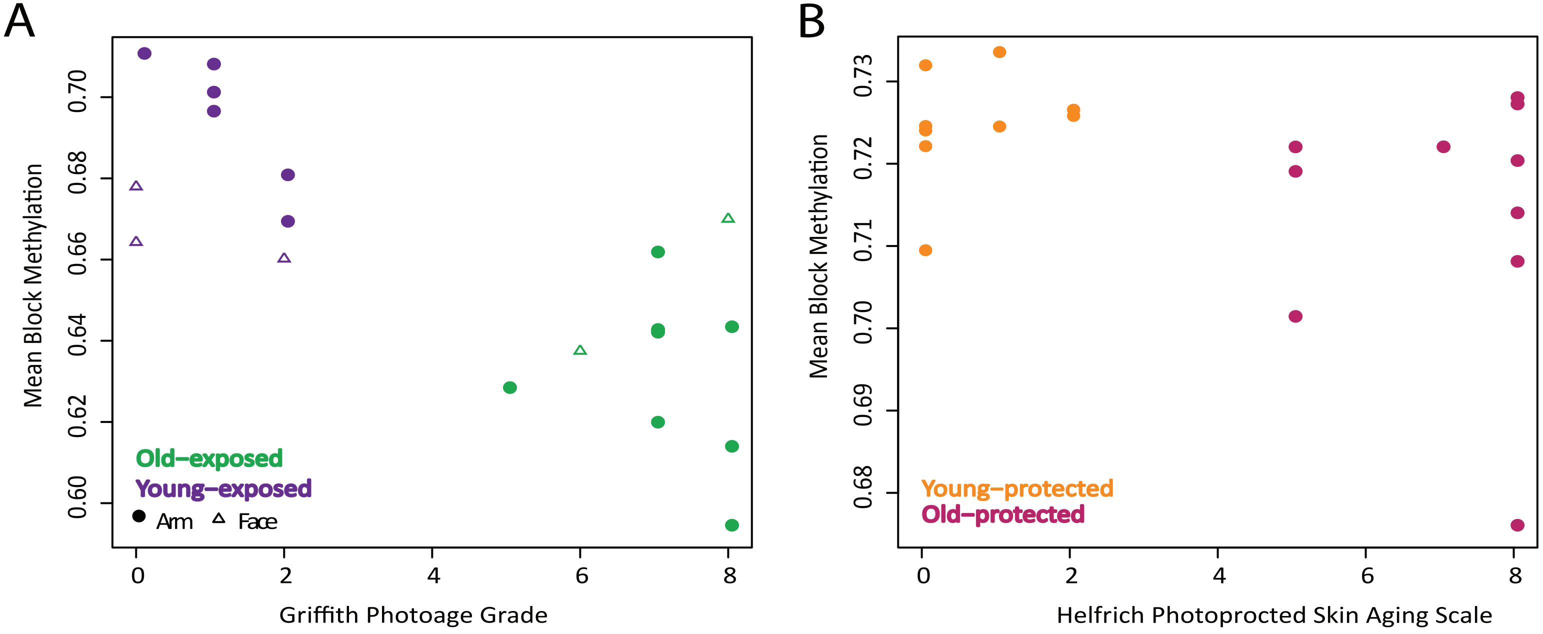

Figure S4. (A) Methylation in sun exposed samples correlates with sun exposed age grade. Shown is mean methylation within blocks identified comparing O-exp and Y-pro epidermis for each sun-exposed epidermal sample versus Griffiths' photoage grade assigned to sample donor. **(B)** Methylation in photoprotected samples does not correlate with photoprotected age grade. Shown is mean methylation within blocks identified comparing O-exp and Y-pro epidermis for each sun-protected epidermal sample versus Helfrich photoprotected age grade assigned to sample donor.
