# Supplementary material for: Age and sun exposure-related widespread genomic blocks of hypomethylation in nonmalignant skin
Source: Genome Biol. 2015 Apr 16;16(1):80. doi: 10.1186/s13059-015-0644-y (PMC4423110; doi:10.1186/s13059-015-0644-y)
Supplement: Additional file 13: Figure S5. — Mean methylation within blocks identified comparing O-exp and Y-pro epidermis versus BMI and smoking status. [file 13059_2015_644_MOESM13_ESM.pdf]

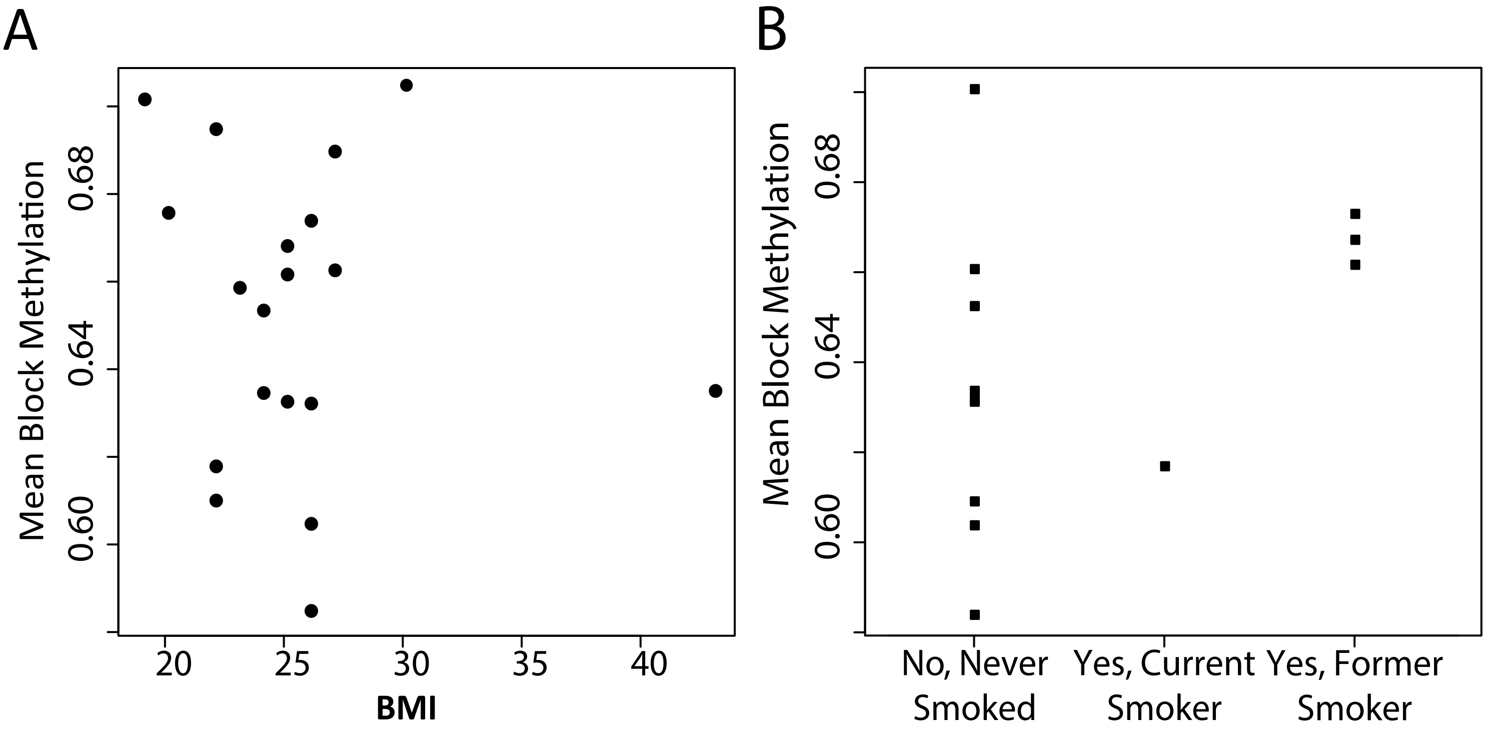

Figure S5. (A) Mean methylation within blocks identified comparing O-exp and Y-pro epidermis for each sun-exposed epidermal sample versus donor BMI. **(B)** Mean methylation within blocks identified comparing O-exp and Y-pro epidermis for each sun-exposed epidermal sample versus smoking status.
