# Supplementary material for: Age and sun exposure-related widespread genomic blocks of hypomethylation in nonmalignant skin
Source: Genome Biol. 2015 Apr 16;16(1):80. doi: 10.1186/s13059-015-0644-y (PMC4423110; doi:10.1186/s13059-015-0644-y)
Supplement: Additional file 15: Figure S6. — Volcano plots and the distribution of P-values for differential expression for the O-exp versus Y-pro, O-pro versus Y-pro and Y-exp versus Y-pro comparisons. [file 13059_2015_644_MOESM15_ESM.pdf]

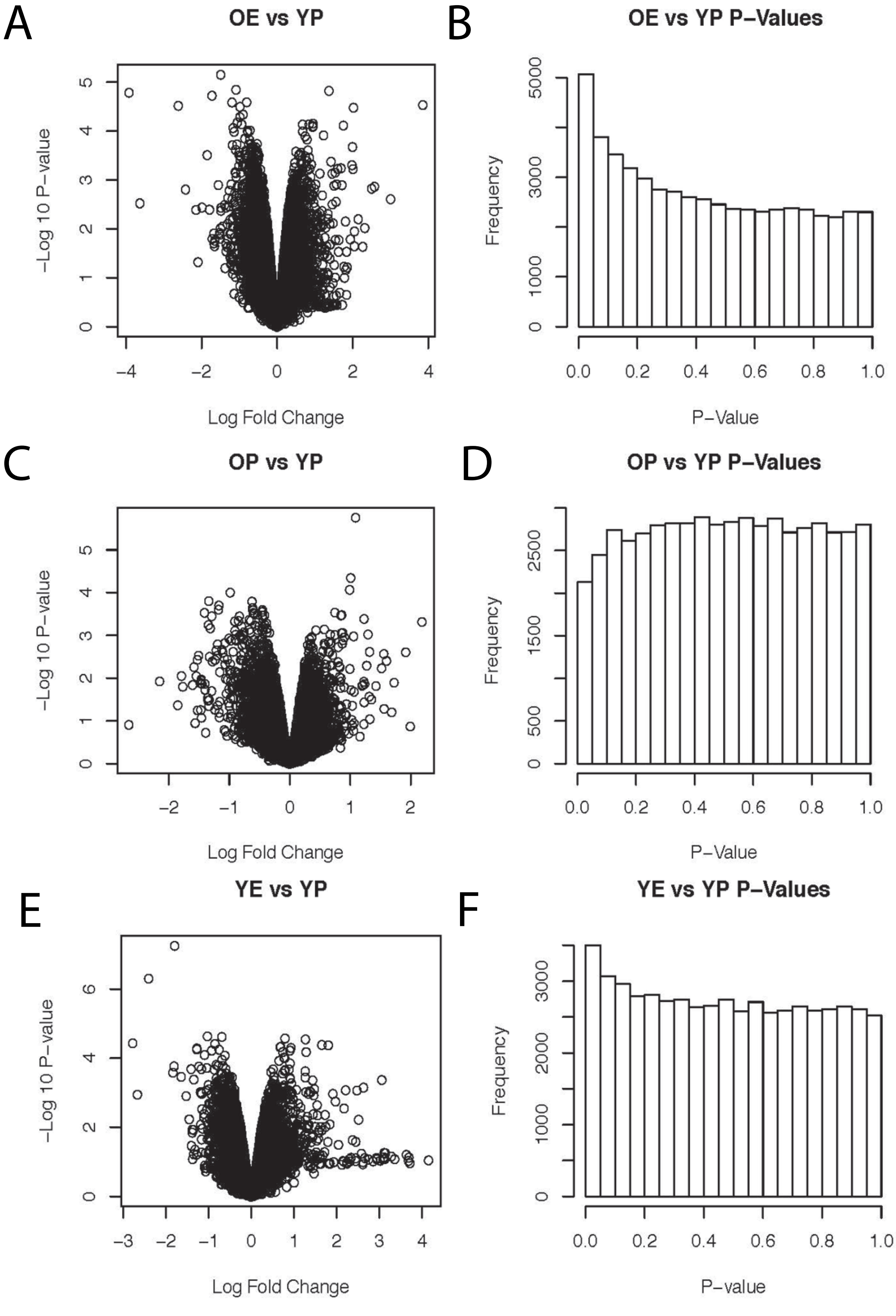

Figure S6: Differential gene expression in epidermal samples with sun exposure and aging. **A)** Volcano plot comparing significance and magnitude of differential expression for the O-exp vs Y-pro comparison. **B)** Distribution of p-values for the O-exp vs Y-pro comparison. **C)** Volcano plot comparing significance and magnitude of differential expression for the O-pro vs Y-pro (Intrinsic Age) comparison. **D)** Distribution of p-values for the O-pro vs Y-pro (Intrinsic Age) comparison. **E)** Volcano plot comparing significance and magnitude of differential expression for the Y-exp vs Y-pro (Exposure) comparison. **F)** Distribution of p-values for the Y-exp vs Y-pro (Exposure) comparison.
