# Supplementary material for: Age and sun exposure-related widespread genomic blocks of hypomethylation in nonmalignant skin
Source: Genome Biol. 2015 Apr 16;16(1):80. doi: 10.1186/s13059-015-0644-y (PMC4423110; doi:10.1186/s13059-015-0644-y)
Supplement: Additional file 16: Figure S7. — Expression levels of melanocyte-specific markers in our sample group. [file 13059_2015_644_MOESM16_ESM.pdf]

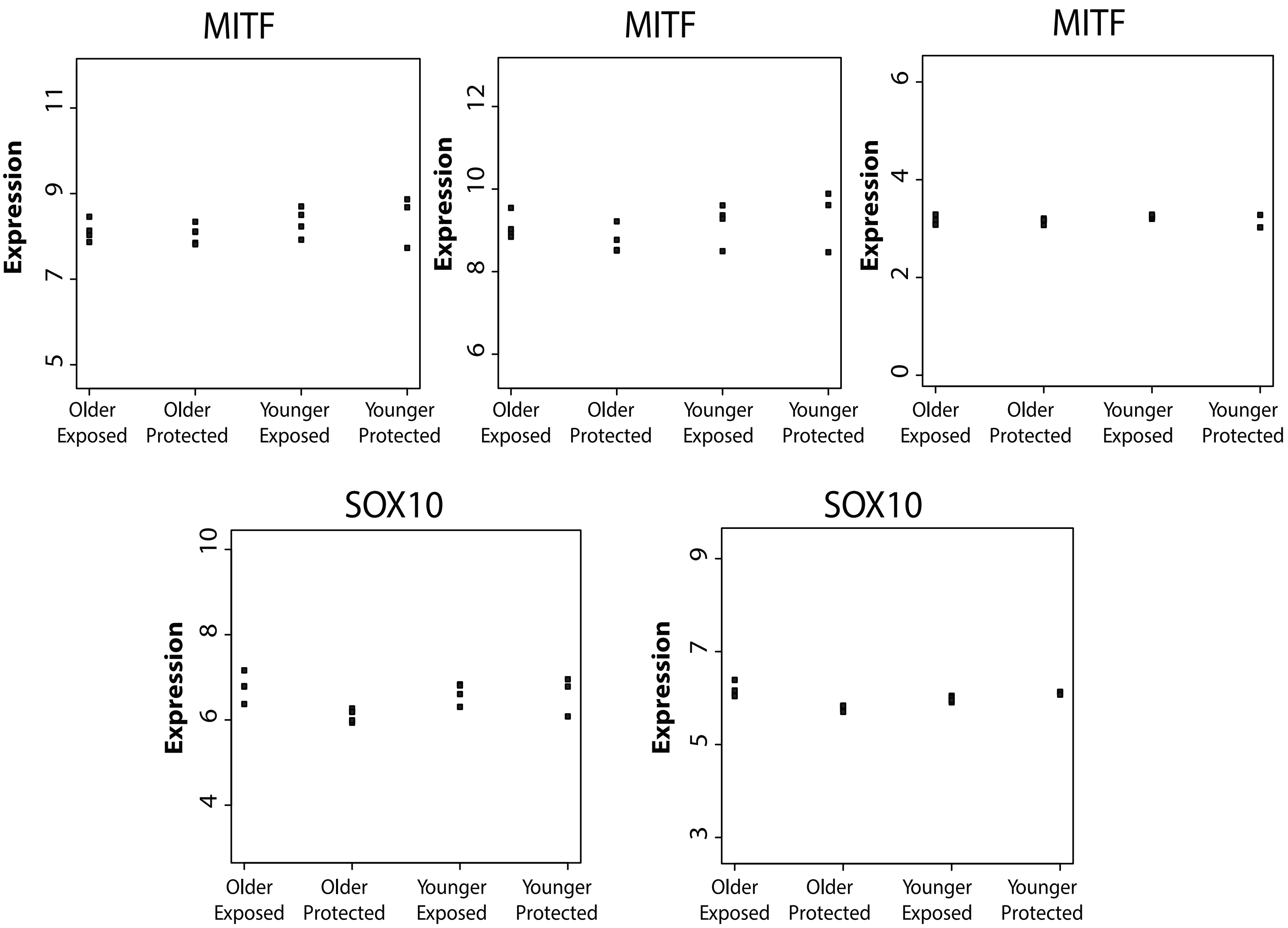

Figure S7: Expression of melanocyte markers is not significantly different with aging and sun exposure status.
A) Log2 expression of melanoctye marker, MITF, measured by 3 affymetrix probes verus age and sun-exposure status for epidermal samples. B) Log2 expression of melanoctye marker, SOX10, measured by 2 affymetrix probes versus age and sun-exposure status for epidermal samples.
