# Supplementary material for: Age and sun exposure-related widespread genomic blocks of hypomethylation in nonmalignant skin
Source: Genome Biol. 2015 Apr 16;16(1):80. doi: 10.1186/s13059-015-0644-y (PMC4423110; doi:10.1186/s13059-015-0644-y)
Supplement: Additional file 17: Figure S8. — Correlation between the methylation age calculated with Horvath’s algorithm and chronological age in dermal and epidermal samples. [file 13059_2015_644_MOESM17_ESM.pdf]

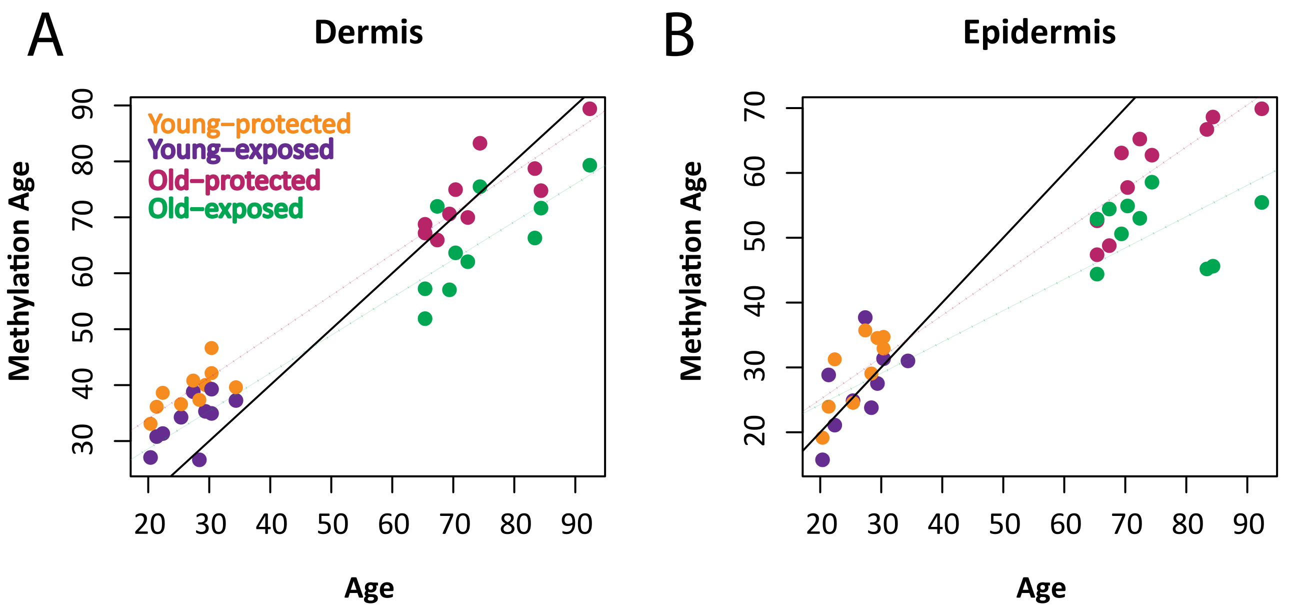

Figure S8. (A) Methylation Age correlates with chronological age in dermal samples. Shown is the methylation age calculated using Horvath's algorithm for each dermal sample versus chronological age for sample donor. (B) Methylation Age correlates with chronological age in epidermal samples. Shown is the methylation age calculated using Horvath's algorithm for each epidermal sample versus chronological age for sample donor.
