# Supplementary material for: Age and sun exposure-related widespread genomic blocks of hypomethylation in nonmalignant skin
Source: Genome Biol. 2015 Apr 16;16(1):80. doi: 10.1186/s13059-015-0644-y (PMC4423110; doi:10.1186/s13059-015-0644-y)
Supplement: Additional file 20: Figure S9. — Demonstrates no age-related methylation changes within the blocks identified comparing O-exp and Y-pro epidermis in three public methylation data sets from peripheral blood and adipose tissue. [file 13059_2015_644_MOESM20_ESM.pdf]

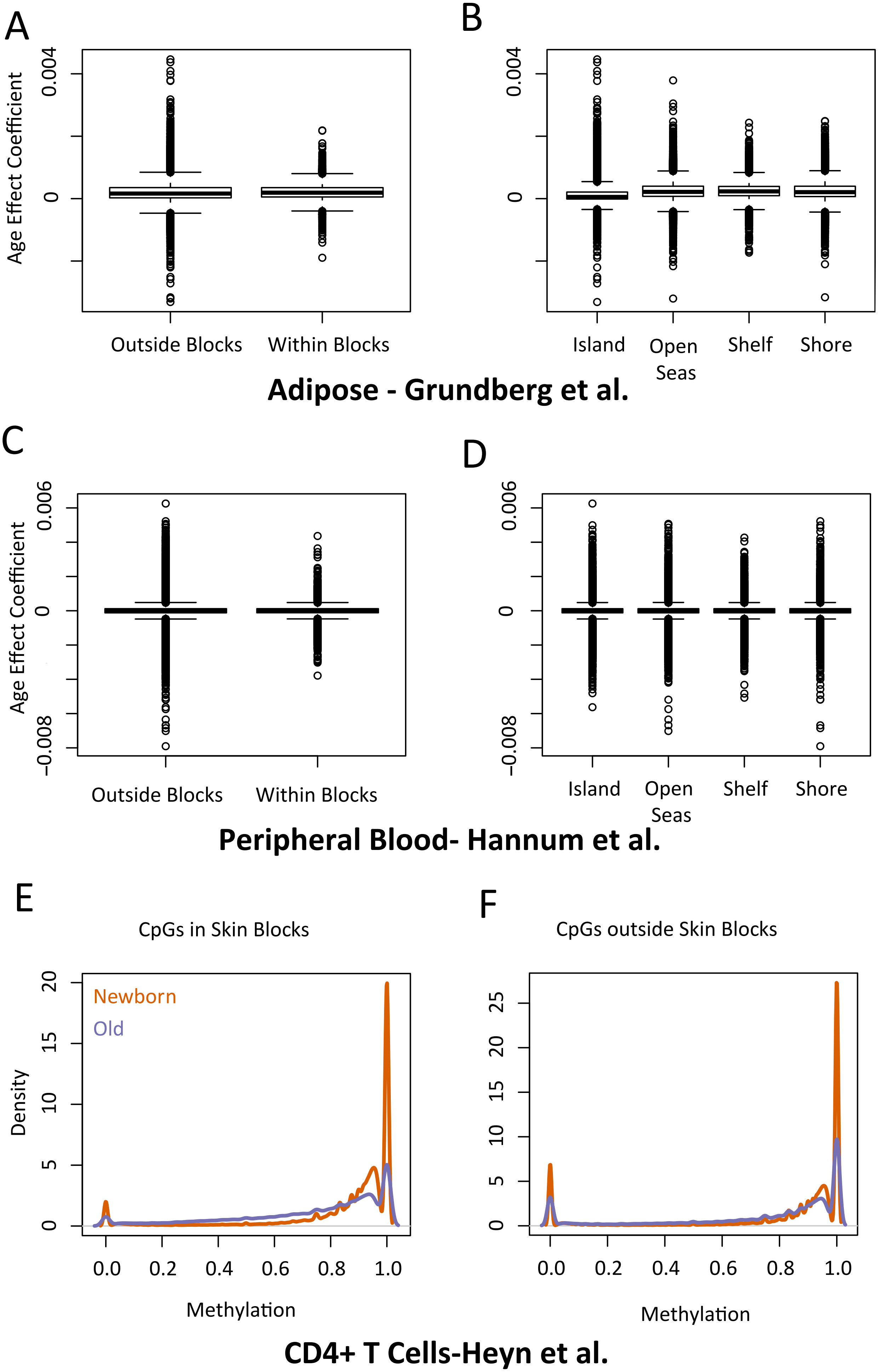
A
B
Age Effect Coefficient
0.004
0
Outside Blocks
Within Blocks
Island
Open Seas
Shelf
Shore
Adipose - Grundberg et al.
C
D
0.006
0
-0.008
Peripheral Blood- Hannum et al.
E
F
CpGs in Skin Blocks
CpGs outside Skin Blocks
Newborn
Old
Density
Methylation
0.0
0.2
0.4
0.6
0.8
1.0
0
5
10
15
20
25
CD4+ T Cells-Heyn et al.

Figure S9. Block methylation in other aging models. **(A)** The age effect coefficient for adipose tissue data from Grundberg et al. for 450k probes within and outside blocks identified comparing O-exp and Y-pro epidermis. **(B)** The age effect coefficient for adipose tissue data from Grundberg et al. for 450k probes in CpG islands, open seas, shores and shelves. **(C)** The age effect coefficient for peripheral blood data from Hannum et al. for 450k probes within and outside blocks identified comparing O-exp and Y-pro epidermis. **(D)** The age effect coefficient for peripheral blood data from Hannum et al. for 450k probes in CpG islands, open seas, shores and shelves. **(E)** Density distribution of methylation from CD4+ T cells from Heyn et al. for CpGs within blocks identified comparing O-exp and Y-pro epidermis. **(F)** Density distribution of methylation from CD4+ T cells from Heyn et al. for CpGs outside blocks identified comparing O-exp and Y-pro epidermis.
